# The impacts of diet on cardiac performance under changing environments

**DOI:** 10.1242/jeb.247749

**Published:** 2024-10-11

**Authors:** Erika J. Eliason, Emily A. Hardison

**Affiliations:** ^1^ Department of Ecology, Evolution and Marine Biology, University of California, Santa Barbara, Santa Barbara, CA 93106, USA; ^2^ Faculty of Science, Kwantlen Polytechnic University, Langley, BC, Canada, V3W 2M8; ^3^ Department of Biological Sciences, University of Pittsburgh, Pittsburgh, PA 15260, USA

**Keywords:** Ectotherm, Food, Heart, Nutrition, Plasticity

## Abstract

Natural and anthropogenic stressors are dramatically altering environments, impacting key animal physiological traits, including cardiac performance. Animals require energy and nutrients from their diet to support cardiac performance and plasticity; however, the nutritional landscape is changing in response to environmental perturbations. Diet quantity, quality and options vary in space and time across heterogeneous environments, over the lifetime of an organism and in response to environmental stressors. Variation in dietary energy and nutrients (e.g. lipids, amino acids, vitamins, minerals) impact the heart's structure and performance, and thus whole-animal resilience to environmental change. Notably, many animals can alter their diet in response to environmental cues, depending on the context. Yet, most studies feed animals *ad libitum* using a fixed diet, thus underestimating the role of food in impacting cardiac performance and resilience. By applying an ecological lens to the study of cardiac plasticity, this Commentary aims to further our understanding of cardiac function in the context of environmental change.

## Teaser

**Summary:** The nutritional landscape is changing, altering diet quantity, quality and options. Altered diets impact cardiac plasticity and function, and thus whole-animal performance and resilience to environmental change.

## Introduction

Environmental conditions (e.g. precipitation, salinity, temperature, pH, oxygen levels, CO_2_ levels, turbidity) are inherently dynamic, varying spatially (local, global) and temporally (diurnal, seasonal, decadal) (Easterling et al., 2000; Frölicher et al., 2018). In addition, both natural and anthropogenic stressors (e.g. heat waves, drought, wildfire, floods) are increasing in prevalence and magnitude, impacting the behaviour, physiology and distribution of organisms across the globe (Buckley et al., 2023; Pörtner and Farrell, 2008; Smale et al., 2019). To survive and thrive in heterogeneous, changing environments, animals can reversibly alter their morphology and physiology via acclimation processes to cope with the new conditions (Seebacher et al., 2015). Food provides the energy and nutrients that animals need to thrive (e.g. move, grow, interact, reproduce) and respond to environmental stressors through physiological acclimation or behavioural responses (Hardison and Eliason, 2024). However, changing environmental conditions are also dramatically altering the nutritional landscape for many animals (Poloczanska et al., 2013). Environmental alterations such as habitat destruction, overfishing, pollution, drought, wildfire, expansion of marine dead zones and rising temperatures impact food availability and the nutritional quality of animal diets, which could profoundly impact animal performance and plasticity.

**Glossary**

**Aerobic scope**

The aerobic capacity for an organism to perform activities above routine maintenance (e.g. locomotion, digestion, reproduction). Calculated as the difference between maximum metabolic rate and standard metabolic rate.

**Cardiac performance**

The capacity of the heart to pump blood, which is determined by numerous factors such as heart rate, stroke volume, synergy of contraction and blood pressure.

**Cardiac thermal limits**

The temperature at which cardiac performance becomes impaired.

**Primary production**

The process of converting inorganic substrates into organic compounds. These organic compounds form the base of food webs.

**Relative ventricular mass (RVM)**

The mass of the ventricle normalized to body mass: RVM=(ventricle mass/body mass)×100.

**Specific dynamic action (SDA)**

The increase in metabolic rate associated with feeding.

**Fig. 1.**
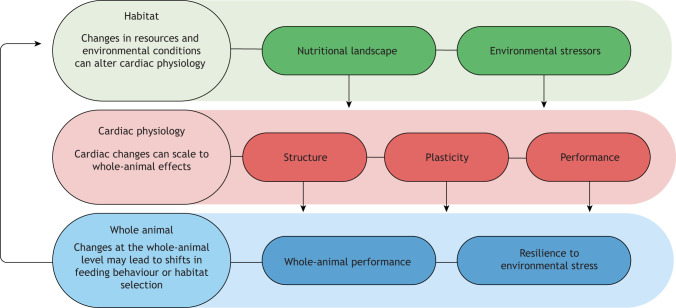
**Conceptual overview of diet and environmental stressor impacts on cardiac physiology and whole-animal performance.** Changes in dietary resources (quantity, quality, options) and environmental conditions (e.g. temperature, hypoxia) impact the structure, function and plasticity of the heart. Given the key role of the heart in transporting oxygen, nutrients and other substances throughout the body, alterations to the heart impact whole-animal performance and environmental resilience. Shifts in whole-animal performance and tolerance limits can have ecosystem-level consequences; for example, via changes in habitat selection or feeding behaviour, thereby changing the nutritional landscape and environmental stressors the animal is exposed to.

The heart is of particular interest because it is critical to support whole-animal performance and environmental tolerance via transport of O_2_, waste, nutrients, hormones and signalling molecules (Eliason and Stecyk, 2020). Diet has great potential to impact cardiac structure, performance and plasticity (Hardison et al., 2021), and thus whole-animal environmental tolerance (Hardison and Eliason, 2024). In turn, shifts in performance and tolerance limits can alter the role animals play in the ecosystem (Fig. 1) and consequently overall ecosystem functioning. Yet, most studies examining animal resilience to environmental stressors feed *ad libitum* with constant, often unnatural diets (Huey and Buckley, 2022). Outside the lab, many animals have the capacity to modify their diet to regulate their nutritional intake, and by doing so, they may be able to improve their performance under suboptimal conditions. In this Commentary, we highlight how we may be underestimating animal plasticity and resilience if we do not consider how nutritional context impacts cardiac performance (see Glossary).

## How is diet changing in response to environmental stressors?

Environmental stressors are changing the nutritional landscape in several ways. First, food quantity (i.e. the amount of available food) varies both spatially and temporally. Consider the seasonal return of salmon species to freshwater habitats across the northern Pacific Rim, which brings huge nutrient influxes to local environments (Naiman et al., 2002). Reductions in salmon returns in recent years have decreased this reliable pulse of nutrients, negatively impacting the flora and fauna that depend on these nutrients (Oke et al., 2020). The global expansion of marine dead zones (severe decrease in dissolved oxygen) has caused mass mortality and changes to community structure (Diaz and Rosenberg, 2008). Ocean warming is predicted to decrease primary production (see Glossary) and marine biomass across all trophic levels (Lotze et al., 2019), and reduce ectotherm body size, a phenomenon known as the temperature–size rule (Atkinson, 1994; Daufresne et al., 2009). Flying insect biomass has declined by ∼75% in Germany over the last 27 years, which is expected to have adverse impacts on ecosystem functioning given their role in pollination, herbivory and as a food source (Hallmann et al., 2017). In addition, both inter- and intra-specific interactions can modify the amount of food consumed. Predators can constrain the capacity of prey to feed, thus lowering the prey's feed intake (Lovegrove, 2000; Nelson et al., 2004). Competition for food also impacts many animals' foraging capacity. Thus, changes to population sizes and food web structure could result in more or less food being available.

Diet options are also changing as the diversity, abundance, size and distribution of plants and animals shift. For example, climate warming is driving reductions in arthropod abundance and food web restructuring in Puerto Rico's tropical rainforest (Lister and Garcia, 2018). In response to the extreme marine heat wave in North America's west coast throughout 2014–2016, known as the Blob, sessile invertebrate abundance and species richness declined while invasive species increased (Michaud et al., 2022). Just a few years later, an unprecedented heat wave in the Pacific Northwest of Canada (British Columbia) and the adjacent USA (Washington, Alaska) in June 2021 killed millions of rocky intertidal organisms but especially impacted the sessile species (e.g. barnacles, mussels) unable to seek thermal refuge (White et al., 2023). Changing ocean environments have led to an increase in global cephalopod (squid, cuttlefish and octopus) populations (Doubleday et al., 2016). In tropical coral reef ecosystems, anthropogenic stressors such as ocean warming, overfishing and extreme weather events have shifted reefs from being coral dominated to algae dominated (Burkepile and Hay, 2006). However, some species are expanding their range into the Arctic (Chan et al., 2019), changing food options.

Finally, diet quality (i.e. the nutritional content of a particular diet) is dynamic and sensitive to environmental perturbation. For example, leaf quality tends to decline with age, decreasing in nitrogen and water content while increasing in fibre and toughness (Mattson, 1980). Drought, atmospheric CO_2_ and temperature can each alter protein and carbohydrate content in plants (Rosenblatt and Schmitz, 2016). Phytoplankton are the key source of omega-3 polyunsaturated fatty acids (n-3 PUFA) in aquatic ecosystems, yet global production of two of the most important omega-3s, eicosapentaenoic acid (EPA) and docosahexaenoic acid (DHA), are expected to decrease by 8.2% and 27.8%, respectively, with a 2.5°C increase in water temperature (Hixson and Arts, 2016). Giant kelp (*Macrocystis pyrifera*) nutritional quality decreased (nitrogen content declined by 18%, while carbon content increased) in response to warming, negatively impacting many organisms that depend on this foundational temperate coastal ecosystem species (Lowman et al., 2022). Given these widespread changes to the nutritional landscape, it is essential to consider the interactions between diet and the environment on animal physiology when evaluating their response to environmental change.

## What choice do animals have?

Animals eat to satisfy their energetic and nutritional requirements for growth and reproduction, for performing various behaviours and to maintain basic life functions. These needs change depending on the environmental context. When something increases an individual's nutritional demand – like how warming raises ectotherm metabolic rates – the animal will compensate by eating more. While this may seem like a simple response, it actually involves a complex and fascinating interplay of signals from the environment, as well as the sensory, gastrointestinal, cardiorespiratory and nervous systems of the animal – ultimately leading to the behavioural response of that animal seeking out and ingesting more food (Blanco et al., 2021; Stubbs, 1999).

In addition to adjusting how much they eat, many animals change their diet selection in response to environmental stimuli. For example, European sea bass (*Dicentrarchus labrax*) alter their macronutrient selection at different water salinities (Rubio et al., 2005), while caterpillars change their macronutrient selection in response to temperature (Lee et al., 2015). Some aquatic omnivores (e.g. zooplankton, amphibians, crayfish, fish and snails) consume more plants relative to prey as water temperature increases (Zhang et al., 2020). In contrast, flies (*Drosophila* sp.) will preferentially eat plants relative to yeast to survive the winter cold (Brankatschk et al., 2018). Herbivorous amphipods prefer algae grown under normal conditions over algae grown in conditions that simulate ocean acidification (Duarte et al., 2016). And several species (e.g. mammals, amphibians, fish, invertebrates), when deprived of a nutrient, will subsequently select for that nutrient when presented with the option later (Raubenheimer and Jones, 2006; Raubenheimer et al., 2009; Kohl et al., 2015). It is remarkable that animals can exhibit adaptive feeding preferences that change in response to environmental stimuli, especially given the complexity of feeding physiology and behaviour. Animals must choose between diets that differ in quality, availability, size, foraging costs, predation risk, competition and habitat, among others. Environmental change can affect all these factors and, at the same time, influence how animals obtain, process, digest and assimilate various nutrients, as well as the functionality of those nutrients in the body. While these interactions certainly make diet selection a challenging topic to study, it is clear that bad diet decisions can lead to nutrient deficiencies, imbalanced nutrition and significant fitness consequences (e.g. impaired growth, reproduction or locomotion) that are exacerbated by environmental change.

**Fig. 2.**
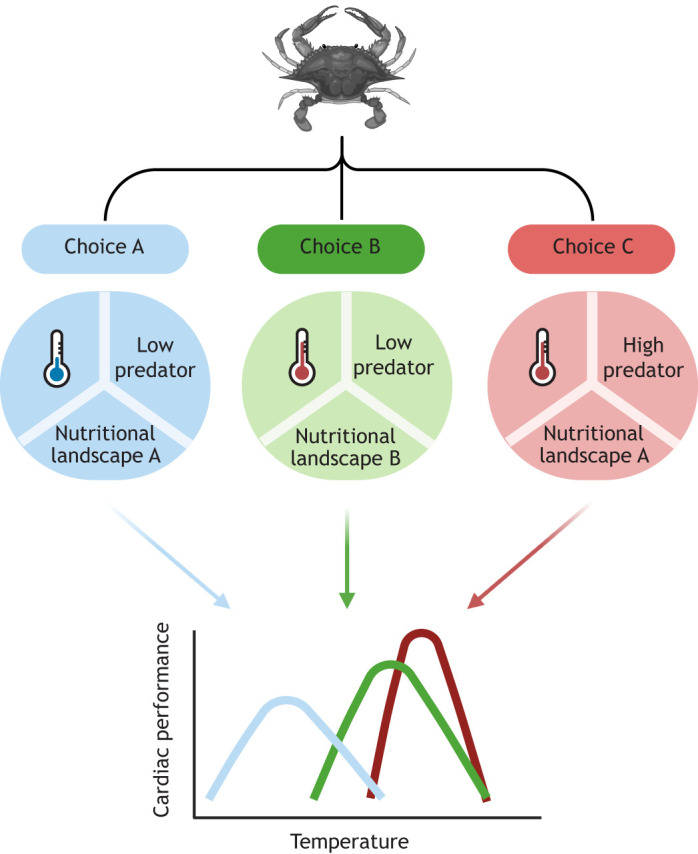
**In heterogeneous environments, mobile animals may select across several habitat choices, with varying physiological outcomes.** In this example, a crab can choose among three habitats that vary in temperature, predation pressure and available dietary energy and nutrients. In turn, each of these habitat choices impacts the shape (height, breadth, position) of the cardiac performance curve, resulting in varying maximum performance and cardiac thermal limits.

Many animals can select different habitats (or microhabitats) to optimize their abiotic (e.g. temperature, salinity, oxygen) and biotic (e.g. nutrients, predators, competition) needs. While the capacity for movement varies across taxa, mobile animals – including everything from locusts to lizards to fish – can move about their environment to regulate their exposure to all sorts of environmental variables, including flow, temperature, oxygen, photoperiod, pH, salinity and predators (Huey and Buckley, 2022; Turko et al., 2023; Careau et al., 2014). Larval animals of sessile species can also undertake habitat selection during settlement (Raimondi and Morse, 2000; Snelgrove et al., 1999). However, trade-offs frequently exist between different habitat choices (Huey, 1991). For example, a habitat with high food availability may also have high predation pressure and suboptimal temperature conditions (Fig. 2). Different habitat combinations will generate different physiological performance curves (Hardison et al., 2023; Fig. 2). Notably, habitat conditions that benefit one physiological trait (e.g. digestion) may not optimize another trait (e.g. locomotion; Hardison et al., 2021). Animals may choose to capitalize on the benefits of heterogeneous environments by performing feeding migrations to mitigate trade-offs. For example, juvenile coho salmon (*Oncorhynchus kisutch*) make feeding forays into cold habitats with abundant food (salmon eggs) but then travel long distances (350–1300 m) to digest their meal at warmer, optimal temperatures for digestion (Armstrong et al., 2013). Given the many opportunities for diet and habitat selection within most animals' lifetimes, many have the ability to adaptively respond to environmental change by making behavioural changes that improve their physiological performance.

That being said, there are limits on how much an animal can adjust their diet to improve their performance, especially in degraded habitats with limited food availability. Other obvious constraints to diet selection are evolutionary ones on diet strategy; for example, an ambush predator will not suddenly become a planktivore when prey is scarce. Less obvious constraints are the abilities of animals to distinguish fitness-impacting differences in prey quality. For instance, vitamin B1 (thiamine) deficiency is on the rise in several fish species (Edwards et al., 2023) that consume prey containing high amounts of thiaminase I enzyme, which degrades thiamine (Baker et al., 2023). Animals suffering from this deficiency cannot distinguish prey that is high or low in thiaminase, leading to impaired cardiac performance, such as reduced maximum heart rate, lower cardiac thermal tolerance and ventricular enlargement (Baker et al., 2023). Overall, diets are determined by several factors which operate on different time scales and range from an individual's feeding behaviour to evolutionary and ecological responses between predators and prey.

## How does diet impact cardiac performance under changing environments?

Many animals frequently encounter variation in environmental conditions (e.g. temperature, hypoxia, salinity), and climate change is increasing the frequency and severity of extreme events (e.g. drought, flooding, heat waves) (Buckley et al., 2023; Easterling et al., 2000; Frölicher et al., 2018). Thus, acclimatizing quickly in response to a novel environmental condition may be just as critical as the overall acclimation capacity. Diet provides a resource reservoir to environmentally challenged organisms. Nutrition has the potential to influence both the capacity and rate of cardiac acclimation to environmental stressors (Hardison et al., 2023). A more nutritious or generalist diet could provide more resources for the heart to rapidly respond to environmental changes (Heard, 2023; Van Baelen et al., 2023). Diet switching in response to novel conditions could also support rapid cardiac plasticity (Hardison et al., 2023). Below, we highlight some of the major features of diet that are likely to influence cardiac structure, performance and plasticity, drawing on examples from mammals and the biomedical literature when ectothermic examples are lacking. While we focus on ectotherms, many of the ideas discussed apply broadly across animal taxa.

### Quantity/energy density

Stressors that raise metabolic rate require animals to take in more fuel by (1) consuming larger or more frequent meals or (2) selecting more energy-dense meals. In addition to eating more, the animal must increase cardiac output to ensure adequate delivery of oxygen and nutrients to demanding tissues (e.g. muscles). When food is scarce, it can impact whether animals can maintain cardiac performance and meet their metabolic demand, and, as a result, their ability to withstand extreme conditions. During short-term and long-term food limitations, animals may divert blood flow to prioritize nutrient and oxygen delivery to essential systems and functions, leading to performance trade-offs. Prolonged food deprivation can also decrease heart size, ventricular glycogen and triacylglyceride content and heart rate (e.g. fishes; Gamperl and Farrell, 2004). However, in some cases, animals are still able to maintain aspects of their cardiac performance even after several weeks of food deprivation. For example, Atlantic cod (*Gadus morhua*) maintained maximum cardiac output after 10 weeks of food deprivation, despite lower heart rate and heart size (Gamperl and Farrell, 2004). Dungeness crabs (*Metacarcinus magister*) had similar heart rates and cardiac thermal limits (see Glossary) in low and high food treatments at current and predicted climate change temperatures (Mclean and Todgham, 2015). Further, overwintering fishes have varied cardiac responses to the cold, sometimes hypoxic, and food-limited waters in winter, either entering a dormant state characterized by depressed metabolism and corresponding decreases in heart rate and cardiac output (Stecyk et al., 2004) or maintaining activity though higher basal metabolism, cardiac output and relative ventricular mass (see Glossary; Cooke et al., 2003; Eliason and Anttila, 2017). Food deprivation can alter both cardiac physiology and animal behaviour, leading to more risky strategies to obtain food. For example, rainbow trout (*Oncorhynchus mykiss*) had lower resting heart rates during food restriction but were more willing to perform risk-taking behaviours during feeding (e.g. to resume feeding following a simulated predator attack) than well-fed fish (Höjesjö et al., 1999). Responses to food limitation, thus, depend on the life history and ecology of the animal in question as well as the severity and duration of the limitation.

While many stressors raise metabolic rates, some (e.g. hypoxia) can have the opposite effect. Many fishes undergo reflex bradycardia while simultaneously increasing stroke volume to maintain cardiac output during hypoxia (Farrell et al., 2009; Gamperl et al., 2017). Feeding raises metabolic rate, which can lead to trade-offs in blood flow between the gut and other systems when environmental oxygen is limited (Axelsson and Fritsche, 1991; Eliason and Farrell, 2014; but see Axelsson et al., 2002). When challenged by exercise following a meal in normoxic waters, some fish, such as European seabass, display higher gut blood flow at low swimming speeds but will divert blood flow away from the gut as swimming speeds increase. In hypoxic water, the seabass allocate the same amount of blood flow to the gut regardless of whether they are fed or fasted, and gut blood flow also decreases as swimming speed increases (Dupont-Prinet et al., 2009). Environmental hypoxia can change how fish allocate their cardiac output, creating trade-offs between exercise and digestion (Gamperl and Farrell, 2004). Thus, even when food is available, animals may not always be able to take full advantage of it (Salin et al., 2016) or may have to ‘defer’ their specific dynamic action (SDA; see Glossary; Dupont-Prinet et al., 2009). For example, several ectotherms cease feeding at extreme temperatures, which may be a behavioural response the animals employ to reduce metabolism during digestion and preserve aerobic scope (see Glossary) for other fitness-enhancing activities (i.e. predator escape; Jutfelt et al., 2021).

Ultimately, food availability may impact environmental tolerance at the whole-animal level through its effects on the cardiorespiratory system. Results have been mixed, showing positive, negative and negligible effects of food restriction on tolerance to extreme temperatures (Lee et al., 2016; Nyamukondiwa and Terblanche, 2009; Rodgers et al., 2019; Woiwode and Adelman, 1992), salinity (Haller et al., 2014; Smolders et al., 2005), pollution (Holmstrup et al., 2010; Smolders et al., 2005), environmental hypoxia (De Boeck et al., 2013; Huhn et al., 2016) and multiple stressors (Cominassi et al., 2020). Aside from its already noted impacts on the cardiorespiratory system, food availability affects energy balance, the optimal conditions for growth, nutrient assimilation, digestion, risk-taking behaviour and microbiome composition (Brett et al., 1969; Brett, 1971; Höjesjö et al., 1999; Huey and Kingsolver, 2019; Kohl et al., 2014; Secor, 2009). This suggests that food amount may interact with other mechanisms discussed throughout this Commentary to influence the physiology of the heart.

### Lipids

The heart beats because of processes occurring across biological lipid membranes (Hochachka and Somero, 2002). Lipids also serve other functional roles in the heart, including energy storage and metabolism (McKenzie, 2001). Variation in dietary lipid composition can lead to differences in cardiac lipid assimilation and metabolism, and, ultimately, membrane composition and oxidative stress. Membrane structural changes can impact ion movement across the membrane, action potentials, cellular respiration and membrane-bound enzyme activities (Hochachka and Somero, 2002). In turn, changes in cardiomyocyte physiology may affect how well the entire heart reacts to environmental perturbation. For example, sturgeon fed a diet high in n-3 PUFA assimilated more PUFA in their hearts and, as a result, were less sensitive to hypoxia during isolated heart trials compared with fish fed a low n-3 PUFA diet (Agnisola et al., 1996; McKenzie, 2001). While the same patterns were not observed in eels, cardiac n-3 PUFA content has also been linked to resilience against tissue hypoxia in mammals (McKenzie, 2001). However, high PUFA diets are associated with greater cardiac lipid peroxidation and oxidative stress, which could leave hearts vulnerable to secondary stressors that also cause oxidative damage (Crockett, 2008; Else, 2017; Hulbert et al., 2017; Lemieux et al., 2011). While countless studies have examined how dietary lipids impact cardiovascular health in humans (e.g. Ding and Rexrode, 2020; Hu et al., 2001; Kuller, 2006), we are only just beginning to unravel how they impact cardiac performance in the context of environmental change.

Although several environmental factors may influence heart function through lipid composition, temperature has received the most attention because of its overwhelming effects on biological rates and membrane performance (i.e. fluidity and phase state; Hazel, 1995; Hochachka and Somero, 2002). Ectotherms can remodel the lipid composition of their membranes to maintain optimal cardiac performance in response to temperature (termed homeoviscous adaptation; Hochachka and Somero, 2002), and this can occur on rapid time scales (e.g. across the tidal cycle in intertidal organisms such as mussels; Williams and Somero, 1996). While there are several mechanisms by which membranes can maintain function across environmental gradients, a common thermal response observed across a wide range of taxa is to exchange membrane phospholipids (fatty acid lengths, headgroup or the degree of unsaturation in the fatty acid tails) or the composition of other membrane lipids (e.g. sphingolipids, sterols; Hazel, 1995; Ernst et al., 2016). For example, ectotherm membrane fluidity is maintained at warm temperatures by incorporating saturated fatty acids (which decreases membrane fluidity), while in the cold, fluidity is maintained by incorporating unsaturated fatty acids (which increases membrane fluidity). One study evaluated the relationship between ventricular fatty acid composition and cardiac thermal performance in an omnivorous marine fish, opaleye (*Girella nigricans*), acclimated to different temperatures and fed different diets (Hardison et al., 2023). They found that fish with higher ventricular PUFA content had improved cardiac performance in the cold, while fish with more saturated fats in the heart had superior cardiac performance at warm temperatures, consistent with homeoviscous adaptation. Other studies have similarly found relationships between cardiac fatty acid profiles and performance. Work on golden grey mullet (*Chelon auratus*) fed high or low n-3 PUFA diets found variation in mitochondrial function (Salin et al., 2021) as well as ventricular force development, cardiac gene expression and metabolism (Vagner et al., 2019). Two salmonids demonstrated negative relationships between cardiac fatty acids (EPA and arachidonic acid) and individual thermal tolerance (Christen et al., 2020). Collectively, this handful of studies suggests that ventricular fatty acid profiles, acquired through diet, can mediate cardiac thermal performance and may be predictive of species resilience to global change (Christen et al., 2020).

### Proteins, amino acids and their derivatives

Dietary proteins and amino acids are essential for the development, growth, health and survival of animals. Some amino acids can be synthesized by a given organism (termed non-essential amino acids), while other amino acids must be obtained from the diet (termed essential amino acids). Beyond serving as metabolic fuel and the building blocks of proteins, amino acids and their derivatives act as signalling molecules, neurotransmitters and metabolic regulators that can profoundly impact cardiac function. For example, adrenaline (epinephrine; which increases cardiac output) is a derivative of phenylalanine and tyrosine; histamine (neurotransmitter) is synthesized from histidine; glutathione (protects cells from oxidative damage) is formed from glycine, cysteine and glutamate (Li et al., 2021). Deficiencies of key amino acids and their derivatives (e.g. taurine, l-arginine, l-citrulline, l-carnitine) have been linked to cardiac dysfunction across taxa (An et al., 2022; Carubelli et al., 2015).

In mammals, the β-amino acid taurine is abundant in cardiomyocytes, acting secondarily to reduce oxidative stress, mediating intracellular calcium homeostasis and regulating mitochondrial protein production (Dixon et al., 2023; Jong et al., 2012). Taurine deficiency is associated with impaired excitation–contraction coupling, cardiomyopathy and heart failure in mammals, suggesting that it likely has negative impacts across broad taxa (Gates et al., 2022). Recent work by Dixon et al. (2023) discovered that cardiac taurine deficiency in brook char (*Salvelinus fontinalis*) led to reduced resting and maximum heart rates and impaired hypoxia tolerance, yet improved critical thermal maximum. Thus, taurine levels are powerful regulators of cardiac function and environmental stress responses (high temperature, hypoxia) in fish (Dixon et al., 2023). More work is needed to evaluate how taurine levels, and the levels of other key amino acids and their derivatives, impact cardiac performance across taxa and environmental stressors.

### Micronutrients and pollutants

Dietary micronutrients play a pivotal role in supporting cardiovascular health. Countless studies have examined how certain minerals, vitamins and antioxidants such as calcium, zinc, magnesium, selenium, vitamins C, D and E, folic acid and coenzyme Q10 are linked with cardiovascular disease in mammalian systems (e.g. Ingles et al., 2020; Narayanam et al., 2021; Rehman and Jianglin, 2022). However, considerably less attention has been devoted to understanding how dietary micronutrients support cardiovascular performance in non-human, non-model systems, particularly in the face of environmental change.

Thiamine (vitamin B_1_) deficiency has been linked to impaired organ function (e.g. reduced immune function, and neurological function), including cardiac failure in humans (DiNicolantonio et al., 2018). Thiamine is a water-soluble vitamin that plays a pivotal role in energy metabolism and generating ATP by acting as a cofactor for multiple enzyme steps in the pentose phosphate pathway and oxidative phosphorylation. Symptoms of thiamine deficiency in the heart include decreased cardiac size, impaired contractility and cardiac failure (Baker et al., 2023). Though there are numerous sources of dietary thiamine (e.g. bacteria, fungi, grains, leafy vegetables, nuts, seeds and fish), thiamine deficiency is becoming a major concern across animal taxa, including mammals, birds, reptiles, fish and bivalves (Baker et al., 2023; Harder et al., 2018). For several salmonid species, thiamine deficiency has been attributed to the consumption of a thiamine-degrading enzyme, thiaminase, which is known to be present in high concentrations in some prey species (Baker et al., 2023). A study on lake trout (*Salvelinus namaycush*) found that fish fed a thiaminase diet for 9 months had enlarged hearts and reduced cardiac performance under warm temperatures (Baker et al., 2023), demonstrating that vitamin deficiency can impair cardiac structure and function and reduce environmental tolerance limits.

Other small molecule compounds obtained in the diet have been shown to be cardioprotective. For example, allicin, an organosulfur substance found in garlic (*Allium sativum*), has antiarrhythmic and anti-arteriosclerosis impacts in some mammals (Banerjee and Maulik, 2002; Prasad et al., 1995). Similarly, fucoidan, a sulfated polysaccharide found in brown algae (*Fucus vesiculosus*), is known to be cardioprotective in mammals, via the activation of antioxidants and suppression of cytokines and nitric oxide-mediated disorders (Thomes et al., 2010; Zaporozhets and Besednova, 2016). These types of compounds are now being considered as supplements to generate enriched diets in aquaculture to improve cardiac function of fish (Hasler et al., 2000; Papadopoulou et al., 2022).

It is alarming to consider that many environmental pollutants (e.g. plastics, pesticides, pharmaceuticals, PCBs, DDT, PFAS) can end up in the diet of animals, adversely impacting their cardiovascular morphology and physiology (Incardona and Scholz, 2017). Further, many essential elements that are necessary for cellular function under low levels can become toxic at higher concentrations. Though many juvenile and adult animals have protection from contaminants via detoxifying enzymes in the digestive tract and liver (Incardona and Scholz, 2017), some compounds have clear cardiotoxicity. For example, selenium is an essential trace element for all vertebrates, serving as an antioxidant and, thus, reducing hypertrophy, protecting cardiomyocytes and decreasing atherosclerosis (Ingles et al., 2020). However, selenium becomes toxic under even moderate concentrations (Janz, 2012). Though selenium is found naturally in the environment (e.g. in black shale, phosphate and coal deposits), anthropogenic activities (e.g. coal-supported power plants, mining, agriculture) are increasing environmental selenium levels. Inorganic selenium can be biotransformed into seleno-methionine and seleno-cysteine by primary producers and microorganisms and then bioaccumulate via trophic transfer from primary producers to secondary consumers to higher order consumers including fish, birds and mammals (Janz, 2012; Pettem et al., 2018). Indeed, dietary seleno-methionine caused cardiotoxicity in both zebrafish (Pettem et al., 2017) and rainbow trout (Pettem et al., 2018). Other contaminants similarly have cardiotoxic effects in animals (e.g. arsenic: Zhao et al., 2019; aluminium: Cantanhêde et al., 2022; triclosan: Wang et al., 2020; cadmium: Liu et al., 2023).

### Microbiome

While the links between animal diets, the microbiome and cardiac performance under changing environments are more circumstantial, there are several lines of evidence that suggest these interactions require close examination. To start, diet is a major source of inter- and intra-specific variation in the gut microbiome (Kohl and Carey, 2016; Trevelline et al., 2019). Additionally, certain aspects of diet and dysbiotic microbiome composition have been linked to cardiovascular disease in mammals (Wang et al., 2011; Chaikijurajai and Tang, 2021). The gut microbiome can convert nutrients in food into circulating metabolites, such as trimethylamine *N*-oxide (TMAO), certain amino acids, bile acids and short chained fatty acids (SCFA), which are associated with cardiovascular function and disease (Chaikijurajai and Tang, 2021). For example, circulating TMAO is associated with atherosclerosis in mice and humans and is generated because of microbial conversion of dietary phosphatidylcholine to TMA (which is then turned into TMAO; Wang et al., 2011). Probiotics and diet shifts can alter microbiome function and, thus, the amount of circulating metabolites (Chaikijurajai and Tang, 2021). Importantly, though, the relationships between microbially derived metabolites and cardiac function differ across taxa. For example, TMAO may be linked to cardiovascular disease in humans but is critically important and highly abundant in elasmobranch plasma because it indirectly counteracts the protein-destabilizing effects of urea, which the animals use as an osmolyte. As a result, it is not clear how these relationships in mammals relate to ectotherms, especially in the context of environmental change.

Environmental change often modifies the microbiome's composition and function, which can affect host phenotypes and health (Trevelline et al., 2019). In some cases, these changes may be bad for the host, leading to inflammation, increased disease risk, nutrient imbalances or impaired digestive performance (Jiménez and Sommer, 2017; Trevelline et al., 2019). Alternatively, a healthy microbiome may provision nutrients or unlock novel phenotypes that aid hosts experiencing environmental stress or food limitation (Henry et al., 2021). For example, inoculation with beneficial microbes enhanced resistance to temperature-induced bleaching in corals (Rosado et al., 2019). Tadpoles reared with wild pond microbes had higher thermal tolerance and reduced risk of heat-induced mortality compared with tadpoles reared in water depleted of microbes (Fontaine et al., 2022). Environmental change may lead to the loss of microbes that are critical to host cardiovascular health or, conversely, an increase in highly tolerant microbes that improve their host's environmental tolerance (Henry et al., 2021; Trevelline et al., 2019). Altogether, the growing body of evidence on the dynamic nature of the microbiome in response to diet and environmental change, as well as its importance in heart health and environmental physiology, demonstrates a clear need to integrate concepts across fields and investigate their combined role across broad taxa.

## Conclusions

When faced with natural and anthropogenic environmental perturbations, animals require energy and nutrients to support cardiac plasticity and performance. However, the nutritional landscape itself changes in response to these same stressors – altering food quantity, options and quality. Variation in dietary energy density, macronutrients, micronutrients, contaminants and the microbiome can all affect cardiac performance and, thus, the capacity for animals to thrive. However, mobile animals, in particular, have a measure of agency – they may be able to adjust their habitat or their diet selection. As a result, diet plasticity could facilitate physiological plasticity. Notably, trade-offs may occur (e.g. the habitat with the best diet may have more predators or suboptimal temperature regimes). Future work should focus on non-model organisms under ecologically relevant conditions to elucidate how diet impacts cardiac structure, function and plasticity. More broadly, across biological disciplines, most studies feed animals *ad libitum* and do not consider natural diets in study design, thus overlooking the potential for diet to improve (or reduce) cardiac performance and whole-animal resilience. We strongly recommend that researchers carefully consider what and how much they feed their animals during experiments, cautioning that failure to do so may lead to erroneous conclusions. Overall, this Commentary highlights that nutritional context is essential when predicting the cardiac response to environmental change.
